# Supplementary material for: On the Structural and Molecular Properties of PEO and PEO-PPG Functionalized Chitosan Nanoparticles for Drug Delivery
Source: Bioengineering (Basel). 2024 Apr 12;11(4):372. doi: 10.3390/bioengineering11040372 (PMC11048302; doi:10.3390/bioengineering11040372)
Supplement: Supplementary file 1 [file bioengineering-11-00372-s001.zip › bioengineering-2879512-Supplementary.pdf]

Supplementary Information

On the Structural and Molecular Properties of PEO and PEO-PPG Functionalized Chitosan Nanoparticles for Drug Delivery

Rejeena Jha [1], Hyrum Harlow [1], Mourad Benamara [2], Robert A. Mayanovic [1]*

[1] Department of Physics, Astronomy, and Materials Science, Missouri State University,

Springfield, MO 65804, USA; rj23s@login.missouristate.edu (R.J.); hih55s@missouristate.edu (H.H.)

[2] University of Arkansas Nano-Bio Materials Characterization Facility, University of Arkansas,

Fayetteville, AR 72701, USA; mourad@uark.edu

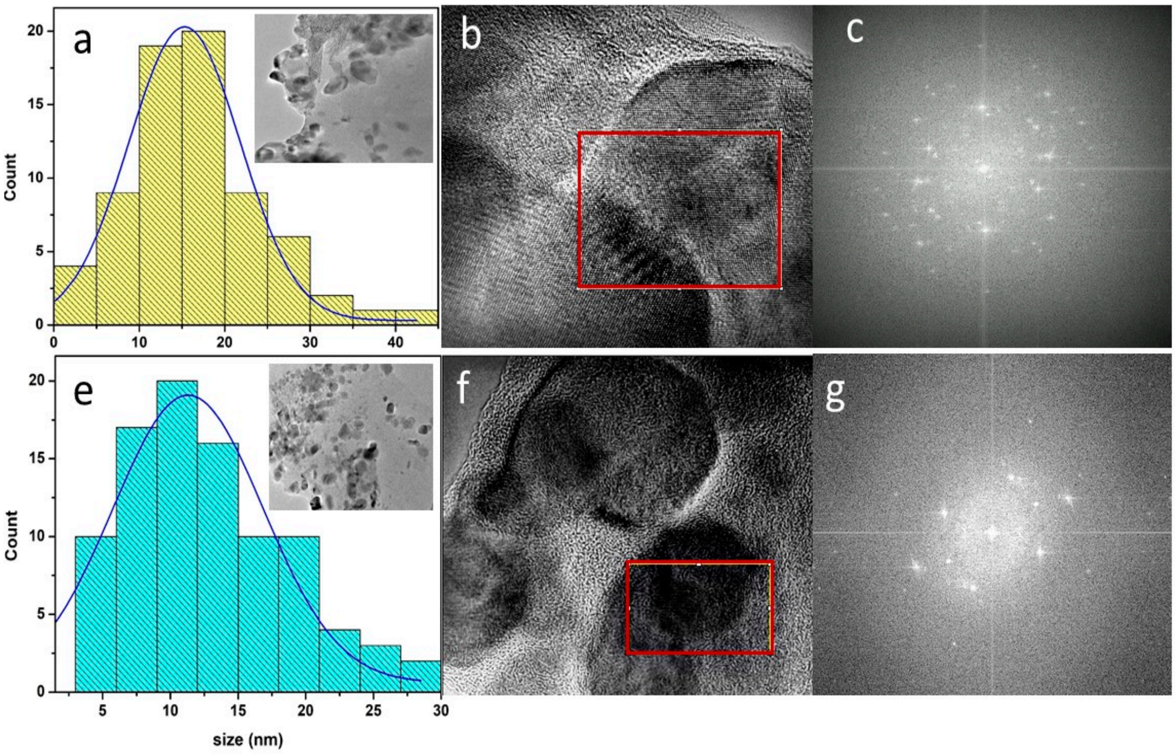

Figure S1. (**a**) Size distribution histogram calculated from TEM data (inset shows a TEM image), (**b**) a HRTEM image and (**c**) a FFT of the region delineated by the rectangle shown in (b) of sample CNP-0.5%; (**d**) size distribution histogram and a corresponding a TEM image shown in the inset, (**e**) a HRTEM image and (**f**) a FFT of the region outlined by the rectangle in e) of sample 1% CNP-PEO.

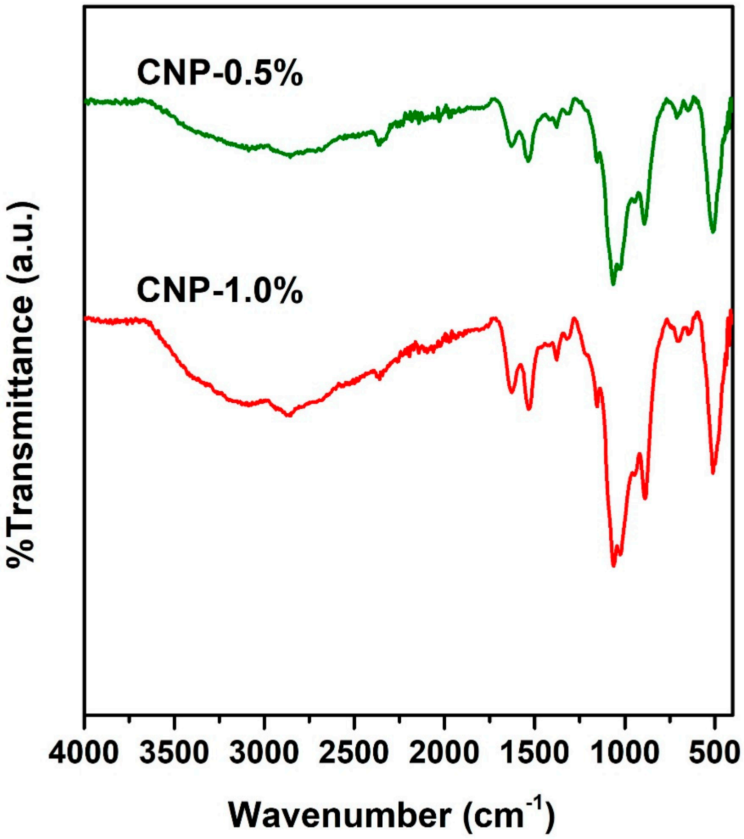

Figure S2. FTIR spectra measured from the CNP-1.0% and CNP-0.5% samples indicating negligible concentration dependence.
